# Acetabular orientation in triple pelvic osteotomy: is intraoperative fluoroscopy reliable?

**DOI:** 10.1007/s00402-022-04568-1

**Published:** 2022-08-10

**Authors:** Daniel Dornacher, Mirco Sgroi, Tobias Freitag, Heiko Reichel, Bernd Lutz

**Affiliations:** Department of Orthopedics, University of Ulm, Oberer Eselsberg 45, 89081 Ulm, Germany

**Keywords:** Triple pelvic osteotomy, Intraoperative fluoroscopy, Hip dysplasia, Hip joint preservation, Surgery

## Abstract

**Purpose:**

In pelvic osteotomies, unfavorable balancing of the anterior and posterior acetabular wall can affect the longevity of the natural joint. This raises the question, whether intraoperative fluoroscopy is sufficiently accurate. The objective was to assess the correlation between acetabular parameters [lateral center edge angle (LCEA), acetabular index (AI), anterior wall index (AWI), posterior wall index (PWI)] acquired on intraoperative fluoroscopic images and postoperative pelvic radiographs and to analyze intra- and interobserver reliability of these parameters.

**Methods:**

A retrospective examination was conducted on 206 consecutive cases (176 patients) after triple pelvic osteotomy (TPO). Every patient received a pre- and postoperative pelvic radiograph in supine position in exactly the same technique. A highly standardized surgical sequence allowed consistent intraoperative fluoroscopic imaging. LCAE, AI, PWI and AWI were measured by an experienced orthopedic surgeon and an orthopedic surgeon in training. Statistics comprised a priori power analysis, Bland–Altman analysis and intraclass correlation coefficient (ICC).

**Results:**

A total of 165 cases were included. ICC between the parameters of the fluoroscopic images and postoperative radiographs was for LCEA: 0.935, AI: 0.936, AWI: 0.725 and PWI: 0.878. Intraobserver ICC for all parameters ranged from 0.953 to 0.989, interobserver ICC from 0.798 to 0.968, respectively.

**Conclusion:**

In the surgical treatment of hip dysplasia by means of TPO, intraoperative fluoroscopic imaging has proven to be reliable and accurate. Intraobserver correlation was excellent for all parameters. The correlation between the intraoperative fluoroscopic images and postoperative radiographs ranged from good to excellent, with the lowest values for the acetabular wall indices (AWI and PWI).

## Introduction

Pelvic osteotomies are a fundamental component in the joint-preserving treatment of symptomatic hip dysplasia. It has been shown that after periacetabular osteotomy (PAO), the native hip can survive at least 3 decades, provided a precise indication and an accurate reorientation of the acetabulum [1]. Particularly, anterior acetabular overcorrection should be avoided since this malorientation has to be regarded as a predisposition for a pincer-type femoroacetabular impingement. This iatrogenic deformity can promote osteoarthritis of the hip and leads to unfavorable results [1–6]. On the other hand, as a consequence of an undercorrection, the load on the acetabular rim and the chondrolabral junction is not reduced sufficiently. In the run-up to a pelvic osteotomy, a comprehensive analysis of acetabular orientation is mandatory to avoid acetabular malorientation. Besides his experienced eye, the orthopedic surgeon has to rely on a set of objectifiable parameters. Recent findings have shown that it is not sufficient to classify a dysplastic hip solely based on the well-established parameters “lateral center edge angle” (LCEA) and “acetabular index” (AI), ignoring the anterior or posterior version of the acetabulum [7]. The recognition of a retroversion sign on a plain pelvic radiograph is basic diagnostics, as there are the well-described “ischial spine sign” and the “cross-over sign”. For a more in-depth deformity analysis, for example, in the workup to a pelvic osteotomy, the anterior- and posterior wall indices (AWI, PWI) provide essential information since they allow to quantify the anterior and posterior coverage of the femoral head [3]. Finally, in the operating room, this valuable preoperative information has to be translated into a physiological acetabular reorientation. For this purpose, intraoperative fluoroscopy is commonly used to guide the surgeon and to assess the final orientation of the acetabular fragment. However, some authors describe a slightly different image of the pelvis in fluoroscopic imaging and recommend to perform a plain pelvic radiograph in the operating room [8]. Currently, the balancing of the anterior and posterior acetabular wall and its implications on the longevity of the natural joint is under discussion. It has been highlighted that particularly the parameters expressing the antero-posterior version of the acetabulum (inter alia PWI) are prone to pelvic tilt or malrotation of the radiograph [9]. This raises the question, whether intraoperative fluoroscopic imaging is sufficiently accurate to justify its application.

To our knowledge, there are no data on this scientific issue for the treatment of symptomatic hip dysplasia by means of triple pelvic osteotomy (TPO). This widely used procedure permits precise and powerful acetabular reorientation. TPO avoids compromising the triradiate cartilage, which has to be pointed out as beneficial in the treatment of acetabular malorientation before skeletal maturity.

The primary objective of this examination is to assess the correlation between acetabular parameters (LCAE, AI, AWI, PWI) acquired on intraoperative fluoroscopic images and postoperative plain radiographs. Furthermore, the aim is to assess intra- and interobserver reliability of the acetabular parameters, measured on the intraoperative fluoroscopic images as well as on the pre- and postoperative plain pelvic radiographs.

## Materials and methods

A retrospective examination on 206 consecutive TPOs was performed. All procedures were performed between January 2016 and December 2019 in our orthopedic department on a total of 176 patients (150 female, 26 males, mean age 26 years, range 9 to 48 years). During this period of time, 30 of the patients also received the procedure on the contralateral side.

The patients were referred to our outpatient department mainly due to the diagnosis of a symptomatic hip dysplasia. Mostly, the patients carried along a pelvic radiograph from the referring physician. Since these images were performed in an inconsistent manner, every patient in the run-up to a TPO received a standardized AP pelvic radiograph in the radiological department of our institution. This comprised supine position, a film–focus distance of 1.15 m, the beam centered between the symphysis and a line connecting the anterior superior iliac spines, both legs fully extended and 15° inwardly rotated. Exactly the same standardized technique was used to obtain the pelvic radiographs in the first follow-up examination after TPO, which was scheduled 5 days after the operation. The radiographs were archived in the picture archiving and communication system of our institution (PACS, GE Centricity Universal Viewer Version 6.0, General Electric Healthcare, Chalfont St Giles, UK).

The TPO was performed in a highly standardized manner, according to the technique described by Tönnis and Kalchschmidt, essentially by one experienced orthopedic surgeon [10]. This allowed a highly standardized intraoperative fluoroscopic imaging. After osteotomy of the sciatic and the pubic bone and before the final osteotomy of the ilium, the c-arm of the fluoroscope was adjusted to resemble the aspect of the preoperative pelvic and acetabular orientation perfectly: after verification of a stable supine position of the patient, the fluoroscope was aligned perpendicularly to the patient. Then, the pelvis was approximated as far as possible to the detector of the c-arm, to obtain a wide overview of both foramina obturatoria, the symphysis and the coccygis. When required, the image was rotated perfectly horizontal. The height–width relationship of the foramina guided the fine-adjustment of the c-arm, often necessitating a slight tilt of the c-arm to compensate for a loss of pelvic inclination, most likely due to a reduction of core muscle tension caused by the general anesthesia. After this calibration at the midline, the fluoroscope was centered over the particular hip joint. Again, the fluoroscopic image of the acetabulum was aligned to the preoperative pelvic radiograph, comparing the aspect of the anterior and posterior wall and—if present—the aspect of the crossing-sign. When no further fine-tuning was needed, TPO was completed with the ilium osteotomy and all fluoroscopic images during the process of acetabular reorientation were obtained with the same adjustment of the fluoroscope.

Intraoperative acetabular orientation was evaluated subjectively. The goals for the orthopedic surgeon were to achieve an acetabular reorientation with a horizontal or slightly upwardly sloping sourcil, to produce a LCEA of around 30°, to resolve acetabular retroversion in a posteriorly dysplastic acetabulum and to avoid anterior overcorrection. At the end of the operation, the fluoroscopic images were transferred to the PACS.

On the preoperative pelvic radiographs, the intraoperative fluoroscopic images and the postoperative pelvic radiographs LCAE, AI, PWI and AWI were measured by an experienced orthopedic surgeon (observer 1, DD). 4 weeks later and after power analysis (see below), the measurements were repeated in 84 randomly selected cases by the experienced surgeon (observer 1, DD) and an orthopedic surgeon in training (observer 2, BL).

### Measurement routine

**Fig. 1:**
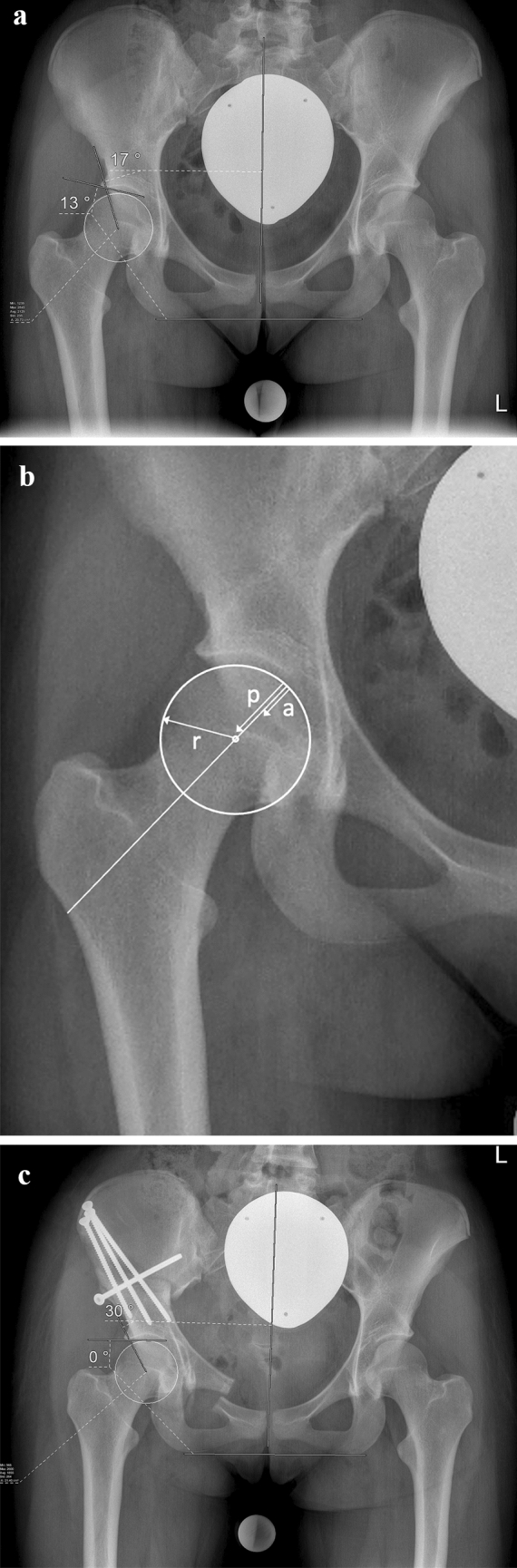
**a** Preoperative radiograph of a 16-year-old female, performed in supine position with the purpose of deformity analysis. In this example, LCEA is 17°, AI is 13°. **b** Magnified view of the right hip from the same radiograph, measurement of the anterior and posterior wall index (AWI, PWI). **c** The measuring procedure was repeated on the intraoperatively acquired fluoroscopic images and the postoperative pelvic radiograph. For the sake of clarity, the measurement lines for AWI and PWI are not presented

After verification of the usability and the relevant landmarks, first of all, the center of the femoral head was estimated from a circle fit to its contour. Then, the longitudinal axis of the pelvis was defined by drawing a vertical line from the processus spinosus of L5 through the middle of the symphysis. The LCEA was measured between the line from the center of the femoral head to the lateral aspect of the sourcil, and the longitudinal axis of the pelvis [11, 12]. Acetabular index was measured between a line connecting the inferior ischial tuberosities and a tangent to the most medial and most lateral aspect of the sourcil (Fig. 1a). For the measurement of the anterior and posterior wall index (AWI, PWI), the circle resembling the contour of the femoral was used again. Lines from the medial contour of the circle to its center (radius r), to the anterior wall (a) and the posterior wall (p) were drawn. The distances were measured along the femoral neck axis. AWI and PWI were calculated as a/r and p/r (Fig. 1b). All parameters were measured on the pre-and postoperative radiography as well as on the intraoperative fluoroscopic image (Fig. 1c).

The following exclusion criteria were defined: pelvic radiographs of patients with a concomitant severe deformation of the femoral head, e. g. due to Legg–Calve–Perthes disease, radiographs of patients with syndromal diseases, radiographs with an unfavorably placed gonad shielding, radiographs with a tilted or mispositioned patient, fluoroscopic images of poor quality, missing or incomplete fluoroscopic images.

### Statistical analysis

Bland–Altman analysis was used to describe the clinical relevance of the findings and to assess the agreement between the postoperative radiograph as a “gold standard” and the intraoperative fluoroscopy. Agreement between the acetabular parameters of intraoperative fluoroscopy and postoperative pelvic radiograph was analyzed by intraclass correlation coefficient (ICC). Intra- and interobserver (observer 1 and 2) correlation was assessed using ICC. The 95% confidence interval (95% CI) was calculated. The values of ICC were interpreted according to the scale described by Cicchetti: less than 0.40: poor, between 0.40 and 0.60: fair, between 0.60 and 0.75: good and greater than 0.75: excellent [13].

The statistical analysis and presentation were performed using SPSS Statistics, Version 26 (IBM, Armonk, New York, United States of America).

For the assessment of intra- and interrater reliability, a priori power analysis indicated a minimum sample size of 84 cases to be measured for an ICC of at least 0.80, two-tailed test, α set to 0.05 (G*Power Version 3.1.9.6).

## Results

After application of the exclusion criteria, a total of 165 cases of TPO were included in this examination. Observer 1 (DD) measured each parameter on all 165 pre- and postoperative radiographs and the intraoperative fluoroscopic images. Mean LCEA of 17° (range 4°–32°) measured on the preoperative radiographs was corrected to a mean LCEA of 28° (range 10°–39°) on the postoperative radiographs (average correction: 11°). Mean preoperative AI of 15° (range 0°–40°) was reduced to a mean postoperative AI of 3° (range − 12°–18°) (average correction − 12°). On the postoperative radiographs, AWI and PWI were assessed 0.39 (range 0.14–0.57) and 1.04 (range 0.63–1.36), respectively.

**Fig. 2:**
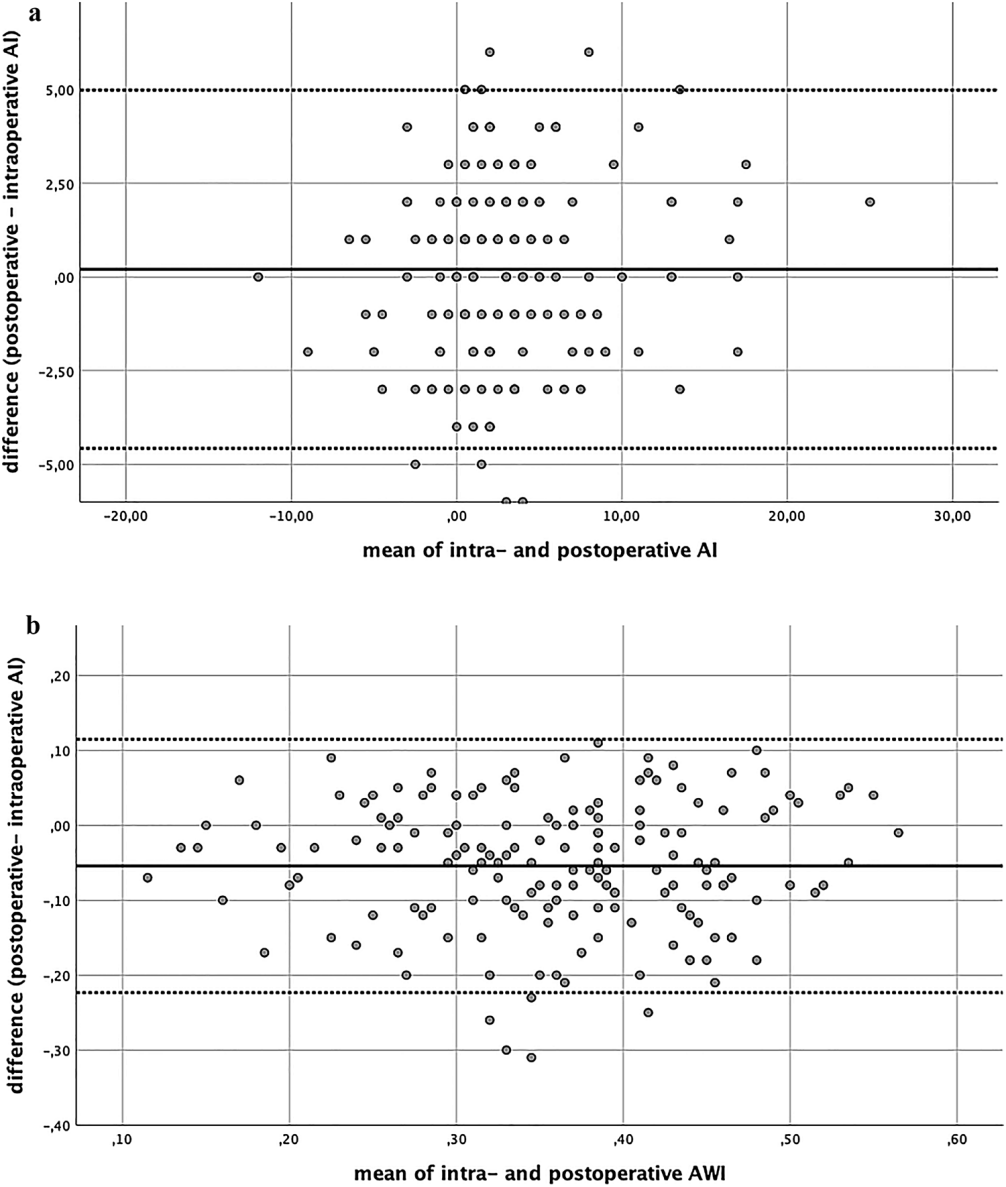
**a** and **b** Bland–Altman diagrams: the *y*-axis displays the differences of the two paired measurements, the *x*-axis shows the mean of the paired measurements. The upper and lower horizontal lines represent the limits of agreement, the horizontal line in the middle the mean difference, respectively. Fig. 2a Bland–Altman diagram of the AI (representing the angular measurements). Fig. 2b Bland–Altman diagram of the AWI (representing the index values). 95% of the data points lie within the limits of agreement (± 2 standard deviation of the mean difference). Mean difference of the AI was calculated 0.20°, of the AWI − 0.053, respectively

Bland–Altman analysis resulted in a mean difference of 0.77° for LCEA (SD: 2.24°), 0.20° for AI (SD: 2.43°), − 0.053 for AWI (SD 0.086) and − 0.055 for PWI (0.085), respectively. The differences of the paired measurements were rather small and the vast majority of the data points ranged within clinically acceptable limits of agreement (Fig. 2). This leads to the assumption that intraoperative fluoroscopic imaging is sufficient in the evaluation of acetabular orientation.

**Fig. 3:**
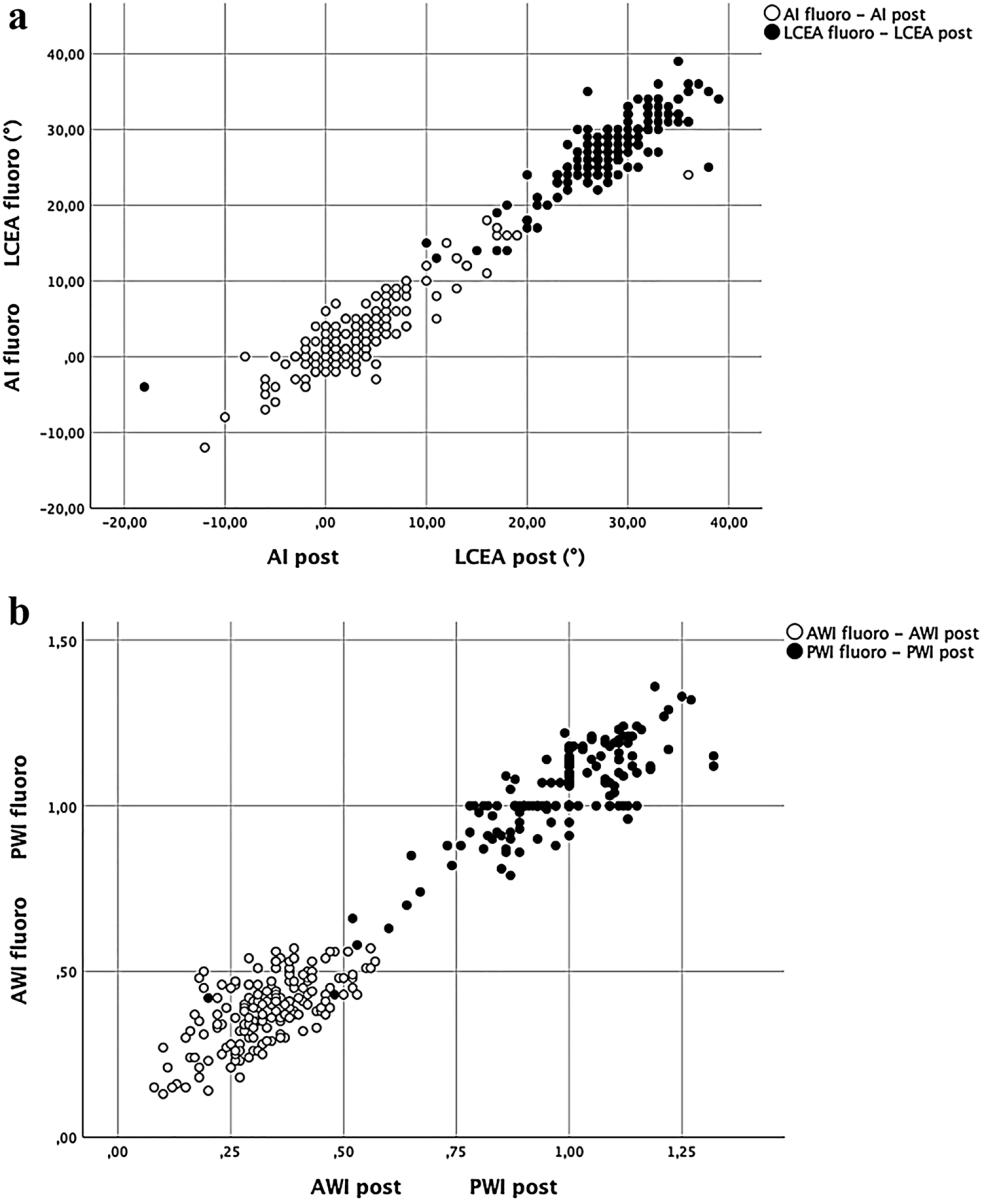
**a** Scatterplot of the parameters LCEA and AI, measured on the intraoperative fluoroscopic images and the postoperative radiographs. For these parameters, correlation between the intraoperative and postoperative imaging was excellent (ICC: LCEA = 0.935, AI = 0.936). **b** Scatterplot of the parameters AWI and PWI, measured on the intraoperative fluoroscopic images and the postoperative radiographs. For these parameters, correlation between the intraoperative and postoperative imaging was good for AWI and excellent for PWI (ICC: AWI = 0.725, PWI = 0.878)

ICC between the parameters of the fluoroscopic images and postoperative radiographs was for LCEA: 0.935, AI: 0.936, AWI: 0.725 and PWI: 0.878. For the sake of clarity, all results including 95% CI are displayed in Table 1 and visualized in Fig. 3. Taking into account the a-priori power analysis, observer 1 (DD) re-read 84 randomly picked cases for the assessment of intra- and interobserver reliability, as well did observer 2 (BL). Intraobserver ICC for all parameters ranged from 0.953 to 0.989, interobserver ICC from 0.798 to 0.968, respectively. The results in detail are presented in Table 2.

**Table 1:** Mean values and ranges for all measured parameters on the pre-, intra- and postoperative images.

Parameter	Preoperative radiograph	Intraoperative fluoroscopic imaging	Postoperative radiograph	Pre- to postoperative radiograph	Intraoperative fluoroscopy to postoperative radiograph	
	Mean, range	Mean, range	Mean, range	Average correction	Observer 1* Average difference; ICC; 95% CI	Observer 2* Average difference; ICC; 95% CI
LCEA	17° (4°–32°)	27.3° (13°–39°)	28° (10°–39°)	11°	0.7° 0.935; 0.908–0.954	−1° 0.787; 0.672–0.862
AI	15° (0°–40°)	2.8° (–12°–18°)	3° (−12°–18°)	−12°	0.2° 0.936; 0.912–0.953	1° 0.875; 0.807–0.919
AWI	0.39 (0.1–0.66)	0.39 (0.14–0.57)	0.33 (0.1–0.57)	−0.06	−0.06 0.725; 0.446–0.844	−0.03 0.675; 0.498–0.790
PWI	0.85 (0.29–1.23)	1.04 (0.63–1.36)	0.98 (0.6–1.3)	0.14	−0.06 0.878; 0.697–0.937	−0,08 0.734; 0.467–0.853

The two columns on the right show the correlation of the intraoperative fluoroscopic images and the postoperative radiographs

**Table 2:** Intra- and interobserver (2 observers) correlation of all parameters

Parameter		Intraobserver reliability			Interobserver reliability	
		ICC		95% CI	ICC	95% CI
Preoperative radiograph						
LCAE	0.985		0.978–0.991		0.960	0.937–0.97
AI	0.989		0.983–0.993		0.968	0.924–0.983
AWI	0.970		0.954–0.981		0.819	0.721–0.882
PWI	0.986		0.979–0.991		0.946	0.917–0.965
Intraoperative fluoroscopic imaging						
LCAE	0.969		0.952–0.980		0.871	0.784–0.921
AI	0.976		0.963–0.984		0.881	0.708–0.941
AWI	0.953		0.927–0.970		0.798	0.689–0.869
PWI	0.974		0.958–0.983		0.910	0.861–0.942
Postoperative radiograph						
LCAE	0.978		0.965–0.985		0.889	0.829–0.928
AI	0.983		0.974–0.989		0.921	0.877–0.949
AWI	0.975		0.962–0.984		0.829	0.737–0.889
PWI	0.975		0.961–0.984		0.856	0.775–0.907

The wall indices (AWI and PWI) showed a slightly but noticeably lower values for ICC in the intraoperative reading. However, intra- and interobserver correlation of all parameters was excellent

## Discussion

The most important finding in this examination was the excellent correlation between the intraoperative fluoroscopic images and the postoperative radiographs, which was reflected by the ICC of LCEA, AI and PWI. The ICC of AWI indicated good correlation. The second most important finding was the excellent intra- and interobserver correlation between all parameters, measured on the pre- and the postoperative radiographs, as well as on the fluoroscopic images.

This study has several limitations: first, this was a retrospective design. Although image acquisition has been highly standardized, it has to be pointed out that the intraoperative fluoroscopic imaging has been performed with three different fluoroscopes. Second, the radiographs which were used in this examination were performed on the fifth day after TPO. It has to be taken into account that the patients still had some physical discomfort to this point in time. This might have altered perfect supine positioning in some cases. However, radiographs on which a relevant malpositioning was recognizable were excluded according to above-mentioned exclusion criteria. Third, patients with a severe deformation of the femoral head were excluded, for example, after Legg–Calve–Perthes disease. For this reason, the results of this examination cannot be transferred to these specific conditions.

As early as 1999, Tönnis and Heinecke described the consequences of acetabular malorientation, in particular the impact of reduced acetabular anteversion on hip associated pain and osteoarthritis [6]. After pelvic osteotomies had been established as a powerful procedure, mainly to correct symptomatic hip dysplasia in the young adult, orthopedic surgeons increasingly became aware of the importance of a precise acetabular orientation. Albers et al. reported on the 10-year survivorship after PAO. The authors pointed out that, amongst others, postoperative acetabular retroversion, excessive acetabular anteversion and undercoverage were linked with a progression of radiographic osteoarthritis [2]. To our knowledge, the retrospective analysis with the longest follow-up evaluating the survivorship of the native hip after PAO was conducted by Lerch et al. The authors overviewed a time period of 30 years after acetabular reorientation, performed in the treatment of hip dysplasia. Postoperative anterior acetabular overcoverage or acetabular retroversion was associated with decreased joint survival [1]. The above-mentioned studies underline the importance of radiographic parameters of the acetabulum in joint preservation surgery and highlight the connection of acetabular orientation with the clinical outcome. In recent years, the introduction of the anterior and posterior wall index enabled a more sophisticated assessment of acetabular orientation. These wall indices allowed to rely on the diagnosis of acetabular retroversion not solely on the existence of a crossing sign or an ischial spine sign. Additionally, these parameters provided a tool for the quantification of anterior and posterior femoral head coverage [3]. In contrast to LCEA or AI, the measurement of AWI and PWI on fluoroscopic images has not been thoroughly reviewed with regard to intra- and interobserver reliability.

To our knowledge, the present examination provides the first results of intra- and interobserver correlation of acetabular parameters after reorientation was performed by means of TPO. The values for intra- and interobserver correlation were throughout excellent for both observers (ICC 0.798–0.986) (Table 2). The distribution of the values for ICC of observer 1 resembled the pattern of observer 2: the highest figures for ICC were calculated on the radiographic images and for the parameters LCEA and AI. Slightly lower figures were calculated for the wall indices, in particular for the AWI on the fluoroscopic images. The close resemblance of the readings of both observers underlines the consistency of the parameters with regard to intra- and interobserver correlation. There are two explanations for the slightly lower values ICC of AWI, particularly in the fluoroscopy: first, the exact location of the anterior acetabular rim—which is crucial for the measurement of AWI—might make the most difficulties on fluoroscopic images. Second, there might be a purely mathematical reason: the calculation of an index with one rather low figure (as in AWI the distance from the contour of the femoral head to the anterior rim), assessed in relation to a rather high figure (distance from the contour of the femoral head to its center) is more prone to error: the greater the discrepancy of the related figures, the more amplification of an imprecision in the measurements.

The most useful finding for the day-to-day clinical practice was the good to excellent correlation between the parameters measured on the intraoperative fluoroscopic images and the postoperative radiographs. The readings of both observers resulted in an ICC of LCEA and AI greater than 0.75, and of AWI and PWI greater than 0.6, respectively (Table 2). The lowest figures for ICC were calculated for AWI, the explanation is the same as for the above-described source of error in the intra-and interobserver readings. Lehmann et al. analyzed, whether after PAO acetabular parameters correlated on intraoperative fluoroscopic images and postoperative radiographs. Amongst others, the parameters LCEA and AI were assessed. The authors reported on an excellent ICC for these two parameters (LCEA: ICC = 0.80, AI: ICC = 0.76) and suggested that intraoperative fluoroscopy is an acceptable tool for the assessment of acetabular correction in PAO [14].

In the present examination, regarding acetabular correction achieved after TPO, the mean values of the radiographic parameters measured on the postoperative radiographs hit the desired target zones. Mean LCEA was 28°, mean AI 3°, these values translate into a sufficient lateral coverage and slightly upwardly sloping sourcil. Mean AWI and PWI were 0.33 and 0.98, matching a physiological anterior and posterior coverage [3]. The rare outliers of postoperative LCEA (range 10–39°) and AI (range – 12 to 18°) occurred on the one hand in excessive dysplasia with a very short sourcil, where an excessive correction would have resulted in an unphysiological force transmission of the femoral head into the fossa acetabuli. On the other hand, the rare outliers occurred as an effect of unwanted overcorrection in the treatment of borderline dysplasia.

For the vast majority, the parameters on the postoperative radiographs indicated physiological correction (Table 1). The overall picture of our results and the current literature makes it possible to state that acetabular orientation can be performed precisely using intraoperative fluoroscopy.

There is a different imaging processes between fluoroscopy and radiography: the plain pelvic radiographs were acquired with the antero-posterior beam centered between the symphysis and a line connecting the anterior superior iliac spines. The radiographic image was produced according to the theorem of intersecting lines. On the other side, the fluoroscopic image is produced with a postero-anterior beam, a shorter film–focus distance and centered closer to the femoral head and the acetabulum. Given the differences in the direction of the beam, the film–focus distance and the centering of the beam, a parallax error has to be assumed. To minimize this potential discrepancy, in this examination, a meticulous calibration of the fluoroscope was performed during the operation. The correct matching of the preoperative pelvic radiograph, acquired in supine position, and the intraoperative fluoroscopic image, almost always necessitated a slight adjustment with a tilt and rotation of the c-arm. The slight compensation for rotation most likely considers the parallax error. This phenomenon might lead to an overestimation of the PWI and potentially to an undercorrection of the posterior wall. The often-needed compensation for the pelvic tilt of 5–10 degrees might take into account the intraoperative reduction of pelvic inclination, presumably owed to the relaxation of the core muscles due to the general anesthesia. In this examination, the direct comparison of the mean values from the intraoperative fluoroscopic images and postoperative radiographs of both observers produced an average difference of no more than 1° for LCAE and AI, for AWI and PWI the average difference was less than 0.03. On the basis of the results of this examination, a tendency towards a relevant over- or underestimation was not recognizable. Correlation analysis between the intraoperative fluoroscopic images and the postoperative radiographs showed excellent values for LCAE, AI and PWI (observer 1: ICC 0.878–0.936). Slightly lower figures were computed for AWI (observer 1: ICC 0.725, observer 2: ICC 0.675), similar to the results of the intra- and interobserver correlation, but still equivalent to a good correlation. These results support the necessity to precisely adjust the c-arm, to match the intraoperative fluoroscopic images to the preoperative radiograph,

The postoperative pelvic radiographs, which were used in this examination, were performed on the 5th day after the procedure. The postoperative radiographs were performed in the exact same technique as the preoperative radiographs. Our standardized follow-up protocol includes a clinical and radiographic control 5 days, 6 and 12 weeks and 1 year after TPO; therefore, we could have opted for a later radiograph. The rationale behind the usage of rather early radiographs was to minimize an error caused by a potential change of correction after several weeks of partial weight bearing. Lehmann et al. conducted an examination comparing radiographic parameters of the acetabulum from intraoperative fluoroscopic images and postoperative pelvic radiographs following PAO. The postoperative radiographs included in their study were performed in a range of 4–57 weeks. The authors assumed that there had been no migration of the acetabular fragment, although this issue had not been observed. This would have been of interest particularly in the late-acquired radiographs. The authors acknowledged that small changes in acetabular position were possible [14].

In this examination, all pre- and postoperative pelvic radiographs were performed in supine position. The first radiographic follow-up was performed on the fifth postoperative day, consistently in all patients in this examination. The supine radiograph allowed an early radiographic examination and avoided an insufficient radiograph due to partial weight bearing 5 days after TPO. Kosuge et al. reported on the influence of patient positioning and imaging technique on the radiological features of hip dysplasia. The authors described that standing causes a change in the pelvic tilt which can alter certain radiological measurements relative to the supine position [8]. The work of Kojima et al. and Trœlsen et al. demonstrated an overestimation of the LCE by 1–2° in supine pelvic radiographs when compared with standing radiographs [15, 16]. In our view, the radiographic and fluorographic imaging, consistently performed in supine position, resulted in a high reproducibility of the imaging, although this aspect was not an object of this examination. Our highly standardized follow-up regimen includes radiographic control after 5 days, 6 weeks, 12 weeks and one year after TPO. The reproducibility of the follow-up imaging will be subject of our future research.

## Conclusion

In the surgical treatment of hip dysplasia by TPO, intraoperative fluoroscopic imaging is reliable and accurate. When acetabular orientation is performed, it is mandatory to adjust the c-arm of the fluoroscope to match the configuration of the acetabulum from the preoperative radiograph. This is necessary, because the technical principles of the imaging modalities differ fundamentally. When this is respected, radiographic parameters of the acetabulum will show good to excellent correlation between the intraoperative fluoroscopic images and postoperative radiographs. Particular attention has to be paid to the balancing of the anterior and posterior wall since this assessment is slightly more susceptible for errors.

## Data Availability

The authors agree to deposit the data that support the findings of this examination. The data have not been uploaded to a public repository yet.
